# Study on the Properties of TiC Coating Deposited by Spark Discharge on the Surface of AlFeCoCrNiCu High-Entropy Alloy

**DOI:** 10.3390/ma17164110

**Published:** 2024-08-20

**Authors:** Ying Wang, Cheng Nie, Shengding Wang, Pan Gong, Mao Zhang, Zhigang Hu, Bin Li

**Affiliations:** 1 School of Mechanical Engineering, Wuhan Polytechnic University, Wuhan 430023, China; ywang0515@163.com (Y.W.); w1664045573@163.com (S.W.); libin_027@126.com (B.L.); 2 State Key Laboratory of Materials Processing and Die & Mould Technology, School of Materials Science and Engineering, Huazhong University of Science and Technology, 1037 Luoyu Road, Wuhan 430074, China; pangong@hust.edu.cn (P.G.); zhangmao@hust.edu.cn (M.Z.)

**Keywords:** electro-spark deposition, high-entropy alloys, microhardness, corrosion resistance, TiC coating

## Abstract

Titanium carbide (TiC) coatings were prepared on the surface of AlFeCoCrNiCu high-entropy alloy blocks using electro-spark deposition (ESD). The microhardness and corrosion resistance of the TiC coatings prepared under different voltage and capacitance process parameters were studied. The research shows that the maximum microhardness of the TiC coating on sample 4 (working voltage of 20 V, working capacitance of 1000 μF) is 844.98 HV, which is 81.5% higher than the microhardness of the substrate. This is because the deposition energy increases with the increase in voltage, and the adhesion and aggregation between the coating and the substrate are enhanced, increasing the hardness of the coating. It is worth noting that excessive deposition energy can increase surface defects and reduce the microhardness of the coating surface. Electrochemical testing analysis shows that the corrosion current density of the TiC coating is the lowest (9.475 × 10^−7^ ± 0.06 × 10^−7^), and the coating impedance is the highest (2.502 × 10^3^ Ω·com^2^). The absolute phase angle value is the highest (about 72°). The above indicates that the TiC coating prepared with a working voltage of 20 V and a working capacitance of 1000 μF has better microhardness and corrosion resistance.

## 1. Introduction

With the continuing exploration of the deep sea, the demand for corrosion-resistant deep-sea materials is increasing [1]. In order to solve this problem, some researchers have introduced fiber-reinforced polymer coatings into composite technology; conducted electrochemical corrosion analysis on steel, low-alloy steel, and stainless steel coated with glass-reinforced polymer; and studied their corrosion properties in simulated corrosion environments. The results show that the corrosion rate of steel coated with glass-reinforced polymer is 8 times and 403 times lower than that of uncoated stainless steel and uncoated low-alloy steel, respectively, indicating that the glass-reinforced polymer coating has excellent corrosion resistance [2]. The emergence of high-entropy alloys in recent years has provided a new direction for researchers to study corrosive materials. High-entropy alloys are composed of five or more central elements, and the atomic percentage of each alloy element does not exceed 35%, which is different from traditional alloys that generally consist of one main element. Based on the particular phase composition of the high-entropy alloys [3,4,5], they have high strength and good corrosion, wear, and oxidation resistance [6,7,8,9]. Many offshore facilities experience corrosion and wear when in contact with seawater or salt spray, leading to premature failure of components [1]. In order to further prolong the service life of the material in a corrosive environment, heat treatment can be performed on the high-entropy alloy material. At a reasonable range of high temperatures, the high-entropy effect will homogenize the microstructure and remove the segregation of elements such as the aluminum-rich, copper-rich, and chromium-rich zone, thereby improving its corrosion resistance [10]. In addition, coatings can be prepared on the surface of corrosion-resistant high-entropy alloys to enhance their service life. ESD technology is one of the most effective methods to improve the surface of metal materials. The coatings it prepares can enhance the substrate’s corrosion resistance, wear strength, wear resistance, and oxidation resistance [11,12,13]. Baysan, E et al. [14] used ESD technology to deposit Fe Al and Ni-Al intermetallic compounds on AISI 304 steel and AISI 316L stainless steel surfaces. The study found that both steel plates’ hardness and corrosion resistance were improved. Wang, J. S et al. [15] used laser cladding and ESD deposition cladding with iron-based alloy powder and WC ceramic carbide on Q235 steel to form a composite coating. The microhardness of the composite coating was analyzed by a microhardness tester. The results show that the highest microhardness of ESD deposition is 1262.9 HV, and the average microhardness is 1151.6 HV, which is higher than the average microhardness of the laser cladding coating, 578.3 HV. The ESD process can also improve the surface-forming quality of materials. Researchers have studied and analyzed the process parameters, surface deposition quality, microhardness, and wear resistance of ESD technology [16]. The results indicate that the ESD process can reduce the surface roughness of the substrate by 2–5 times, and the microhardness and wear resistance of the coating are improved by 2–4 times compared with the substrate.

Current research shows that using TiC coatings can effectively enhance the comprehensive performance of components. Researchers have long studied TiC coatings. For example, titanium carbide coatings were deposited on Si substrates using the pulsed laser deposition method, and the effect of deposition temperature on the mechanical properties of the TiC coatings was studied. Research has found that as the deposition temperature increases, the hardness of the coating shows a significant upward trend [17]. Masaoka, H et al. [18] prepared TiC coatings using a gas mixture of titanium tetrachloride, H_2_, methane, and acetylene via radio frequency plasma-enhanced chemical vapor deposition. They studied the dependence of substrate temperature on the Ti/C ratio. Research has found that TiC thin films of methane have a higher hardness (1150 Hv) than those of acetylene thin films. These early research results provide an excellent reference value for the in-depth study of TiC coatings. Researchers have recently used methods such as laser cladding, high-temperature carbon fixation, and electrical discharge deposition to prepare TiC coatings. Zhang W et al. [19] used a carbon layer converted from an organic carbon solution as a carbon source to prepare TiC coating on the surface of 316L stainless steel through a molten salt disproportionation reaction. The prepared TiC coating was smooth and dense. Electrochemical impedance testing found that the TiC coating was consistently more corrosion-resistant than the 316L stainless steel substrate. Using TiC and Ni60 mixed powder as raw material, Cai, Q et al. [20] prepared composite coatings on the surface of Ti6Al4V alloy with laser cladding technology. The effect of the TiC content on the wear resistance of the coating was studied, and it was found that the coating’s wear resistance and bending strength decreased with the increase in TiC content. In addition, some researchers have used high-temperature carbon fixation technology to prepare and characterize the TiC coating on the surface of Ti15Mo alloy with graphene as a carburizing agent. The results show that the maximum surface microhardness of the treated TiC coating can reach 1221.5 HV, which is 3.93 times that of the original Ti15Mo alloy, and the carburized Ti15Mo alloy shows better tribological properties [21]. Mertens, E et al. [22] deposited TiC and WC coatings on the surface of AISI M2 high-speed steel with an ESD deposition process. The coating surface was tested for wear and electrochemical corrosion and compared with untreated AISI M2 steel. The research shows that a TiC coating can improve corrosion resistance more effectively than a WC coating. TiC has shown excellent corrosion resistance and conductivity as a coating material, providing broad application prospects for the surface treatment and modification of alloys. However, compared with surface treatment techniques such as laser cladding, molten salt disproportionation reaction, vapor deposition, and high-temperature carbon fixation, the electric spark deposition process in the preparation of TiC coatings has advantages such as low energy input, simple operation, fast cooling rate, and green environmental protection [23,24,25,26]. However, for using electrical discharge deposition technology, the current theory needs more research on preparing TiC coatings on high-entropy alloy surfaces.

Therefore, this article mainly uses electric discharge deposition technology to deposit TiC coatings on the surface of AlFeCoCrNiCu high-entropy alloy and systematically studies the influence of process parameters on the hardness and corrosion resistance of the coating in detail. The microhardness of the TiC coating was studied using a digital microhardness tester, and the morphology characteristics of the TiC coating were characterized and analyzed using scanning electron microscopy and an electron probe. The corrosion resistance of the TiC coating was studied using electrochemical testing.

## 2. Materials and Experiments

### 2.1. Preparation of Matrix

The high-entropy alloys mentioned in this article are usually prepared using arc-melting technology and are typically melted 3–4 times to achieve chemical homogenization of the alloy. The matrix material used in the experiment is made of high-purity (purity > 99.99%) bulk heavy-metal powders of Al, Fe, Co, Cr, Ni, and Cu as raw materials. AlFeCoCrNiCu high-entropy alloy blocks were obtained by melting three times in a WK-II non-consumable vacuum arc furnace, and the chemical composition after preparation is shown in Table 1. Then, the melted AlFeCoCrNiCu high-entropy alloy was processed into a matrix with dimensions of 20 mm × 6 mm × 3 mm on an online cutting machine. Before sedimentation, we sanded the high-entropy alloy substrate with sandpaper, polished it with a polishing paste, cleaned impurities with alcohol, and blow-dried it for later use.

### 2.2. Preparation of Coatings

**Figure 1:**
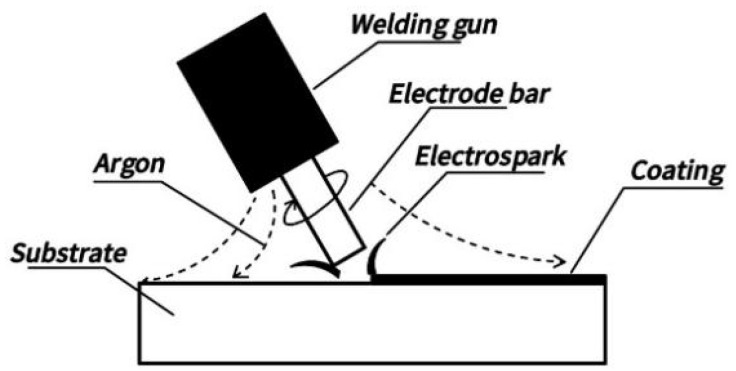
Schematic diagram of the preparation process for electric spark deposition coating.

The electrode rod is made of commercial TiC (purity ≥ 99.5%) metal rod with a size of Ø 6 mm × 30 mm. The deposition experiment was conducted using a DJ-2000 adjustable-power metal surface repair machine, and the deposition schematic is shown in Figure 1. During the experiment, argon gas protection was used, with an argon gas flow rate set at 10 L/min and a deposition time of 1.5 min/cm^2^. The deposition gun was operated at a speed of 2000 r/min and 100% vibration frequency. Six different TiC coatings were prepared by changing the working voltage and working capacitance, as shown in Table 2.

### 2.3. Characterization of Coating Microstructure

The coated sample deposited on the surface of AlFeCoCrNiCu high-entropy alloy was cut into 20 mm × 6 mm × 3 mm samples, and the cross-sections of the substrate and coating joints were ground with 400#, 800#, 1000#, 1500#, and 2000# sandpapers, sequentially. They were polished with diamond grinding paste with a particle size of 2.5 μm on a polishing machine, and finally corroded with nitrohydrochloric acid (HCl:HNO_3_ = 3:1) for 5 s. The surface morphology of the coating and the cross-sectional morphology of the interface between the substrate and the coating were analyzed using an electron probe (EPMA-8050 Shimadzu Corporation, Kyoto, Japan). A wavelength-dispersive X-ray spectrometer was used on the device to perform line scan composition analysis on the cross-section of the interface between the substrate and the coating.

### 2.4. Performance Testing of TiC Coating on AlFeCoCrNiCu High-Entropy Alloy Surface

**Figure 2:**
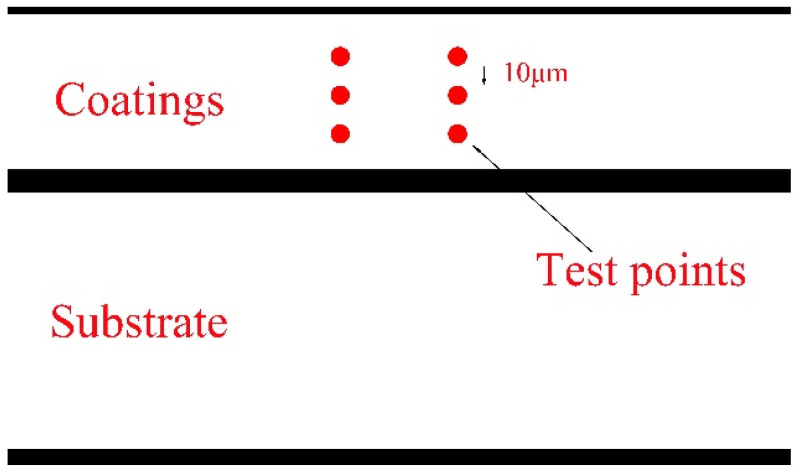
Schematic diagram of microhardness test on TiC coating section.

The microhardness of the coating section was measured using a digital microhardness tester (HVS-1000, JEOL Corporation, Tokyo, Japan) to ensure the accuracy of the experimental data, following this procedure: take 3 points from the surface of the coating to the depth of the substrate, with a distance between the points of 10 μm, and take 2 columns of 6 points in parallel to measure the microhardness, as shown in Figure 2 below. The load loaded in the experiment was 200 g, and the holding time was 10 s. After discarding the maximum and minimum values, the average value was taken to obtain the final hardness value. Then, a vertical chart of cross-sectional hardness was drawn to compare the microhardness values of coatings under different processes.

The corrosion performance of the coating was analyzed using a potentiodynamic polarization curve and electrochemical impedance spectroscopy. The experiment used a multi-channel electrochemical workstation (CS310X, Wuhan Kesite Instrument Co., Ltd., Wuhan, China) and a three-electrode system for corrosion testing. The working electrode was a TiC coating surface deposited on the AlFeCoCrNiCu high-entropy alloy surface and an AlFeCoCrNiCu high-entropy alloy substrate surface, the reference electrode was a saturated calomel electrode (SCE), and the auxiliary electrode was a platinum plate. The electrolyte used in the experiment was a 3.5% sodium chloride aqueous solution (at room temperature), and the container for the corrosive solution was a three-electrode corrosion pool.

The electrochemical corrosion testing process was performed as follows: The first step is to perform cathodic reduction on the working electrode for 3 min to remove surface oxides. Step 2: Conduct an open circuit potential test on the sample for 1 h. Step 3: After the open circuit potential stabilizes, test the impedance of electrochemical impedance spectroscopy with parameters of AC amplitude of 10 Mv and testing frequencies of 10^−2^ to 10^5^ Hz. Step 4: Test the potentiometric polarization curve with parameters of initial potential −0.5 V, termination potential 1.5 V, and scanning speed 1 m/s. Each experiment was repeated three times to ensure the accuracy of the data. The impedance process was simulated using a simulation circuit, and the resistance Rs in the electrolyte, the equivalent polarization resistance Rp in the electrode bilayer, and the capacitance CPE in the circuit were analyzed. The Tafel dynamic potential polarization curve was analyzed to obtain the corrosion potential, corrosion current, and corrosion current density.

## 3. Results and Discussion

### 3.1. Microhardness of TiC Coating on AlFeCoCrNiCu High-Entropy Alloy

**Figure 3:**
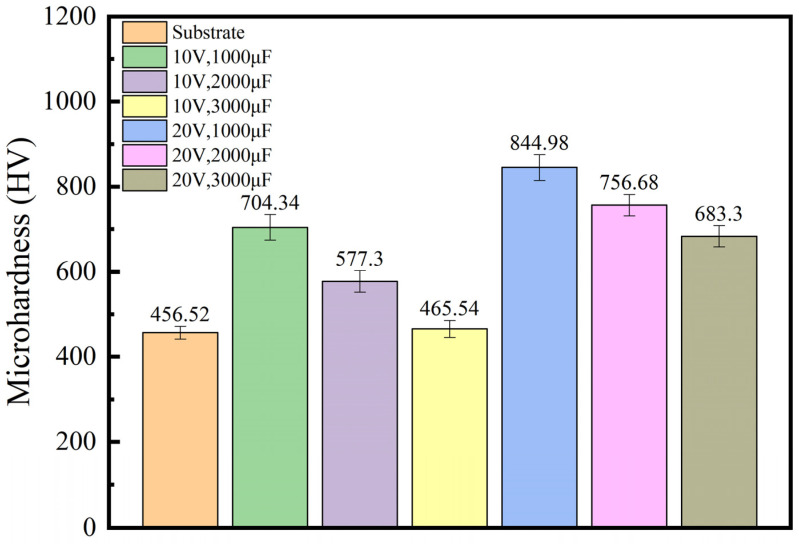
Microhardness of coating cross-sections deposited under different substrate and process parameters.

Figure 3 shows the microhardness values plotted at the cross-sections of six coatings and substrates into a tree diagram. From Figure 3, it can be seen that the cross-sectional hardness of the coating is greater than that of the substrate. Research has shown that TiC coatings prepared by electrical discharge deposition technology can effectively improve the surface hardness of the substrate. Wu, H et al. [27] prepared high-entropy alloy coatings with FeNiCoCr as the substrate, directly added TiC (D-TiC), and in situ added TiC (I-TiC) using laser cladding technology. The average microhardness of the three high-entropy alloy coatings and substrates (316 ss) was studied. The study showed that the average microhardness of the three high-entropy alloy coatings was 68% to 99% higher than that of the substrate, and the microhardness of the coating containing TiC was significantly improved compared with the coating without TiC. X-ray diffraction analysis of the TiC coating found that TiC caused severe lattice distortion, indicating that the presence of TiC led to typical solid solution strengthening of the coating. The melting of large-sized atomic Ti can explain this strengthening into the FCC matrix. However, lattice distortion plays a vital role in resisting material plastic deformation, and the mechanical properties of composite coatings are effectively improved as a result. In addition, Xiao, M et al. [28] achieved hardening, densification, and toughening effects by simultaneously introducing dual-sized TiC particles into TiC/HEA composite coatings. The high microhardness of the coating is due to the hardening effect generated by the dispersion of dual-sized TiC particles in the HEA phase, while the low porosity is due to the in situ shot peening effect and the increased melting degree of TiC ceramics.

Comparing the microhardness of TiC coatings prepared under six different process parameters, it was found that the average microhardness of the TiC coatings prepared at a working voltage of 20 V was 761.65 HV, which is higher than the average microhardness of TiC coatings prepared at a working voltage of 10 V, which was 582.39 HV. This indicates that TiC coatings’ microhardness increases with the increasing working voltage. This phenomenon is related to sedimentary energy. In fact, as the deposition energy increases, the adhesion and aggregation of the coating dominate, leading to an increase in the microhardness of the coating. From Figure 3, it can be seen that the microhardness of the TiC coating decreases with an increase in the working capacitance. This is because when the working voltage is constant, the larger the working capacitance, the greater the pulse energy. According to the law of diffusion, the diffusion coefficient of elements is affected by temperature. Therefore, when the electrode rod is deposited under high capacitance, the high temperature generated by high-frequency vibration friction will accelerate the diffusion of elements, thereby affecting the hardness of the coating and the adhesion between the coating and the substrate. Moreover, as the deposition capacitance increases, the energy of the electric spark discharge increases and the temperature rises. When depositing TiC coating in the atmosphere, high deposition capacitance will cause oxidation of the TiC coating on the substrate surface, resulting in the production of TiO_2_ in the coating. Moreover, with the increase in capacitance and output voltage, the oxidation of the TiC coating intensifies, and the oxidation product TiO_2_ increases, resulting in a decrease in the hardness of the TiC coating.

### 3.2. Surface Morphology Analysis of TiC Coating on AlFeCoCrNiCu High-Entropy Alloy Surface

**Figure 4:**
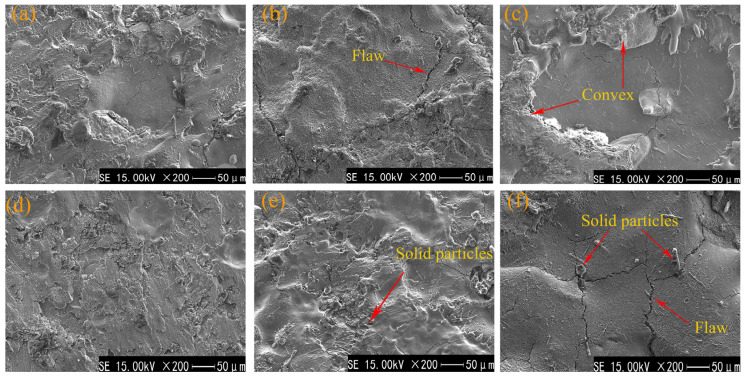
Surface macromorphology of TiC coatings. (**a**) Sample 1; (**b**) Sample 2; (**c**) Sample 3; (**d**) Sample 4; (**e**) Sample 5; (**f**) Sample 6.

In order to study the macroscopic morphology of the TiC coating surface, an electron probe was used to analyze the surface morphology of the coating; as shown in Figure 4, the various defects contained in the macroscopic morphology of the coating surface can be divided into two broad categories: cracks in the coating and stacking of metal droplets. In Figure 4c,e, there is a noticeable stacking of metal droplet materials. In the direction indicated by the arrow in Figure 4c, there appears a “pit surge” that is low in the middle, relatively flat, and high around. This is due to the flow obstruction caused by cooling at the edge, resulting in the stacking of droplet materials. In the discharge channel, the melted electrode material rushes toward the surface of the substrate material with high kinetic energy due to the action of the electromagnetic field. Finally, it forms under the surface tension and cooling. In Figure 4e, it can be seen that the coating surface has a “sputtering-like” pattern morphology with many sputtering points arranged continuously. Many teardrop-shaped tiny droplets appear at the protruding sputtering points in the coating. This is because the molten TiC electrode melted during the deposition process and deposited on the surface of the AlFeCoCrNiCu high-entropy alloy substrate in the form of sputtering or even explosion during the electric discharge process, resulting in the separation and rapid solidification of a small amount of sputtered large and small droplets. According to Formula (1),

(1) $$E=\frac{1}{2CU^{2}}$$

In the formula, *E* represents the pulse energy (unit J), *C* represents the capacitance at deposition (unit F), and *U* represents the voltage at deposition (unit V). It can be seen from Formula (1) that the deposition energy of ESD is proportional to the square of the capacitance and voltage. Noticeable cracks in the coating can be seen in Figure 4b,f. The reason for the cracks is that with the increase in capacitance or voltage, the pulse energy also increases, and the heat input to the matrix material increases during the deposition of ESD. When the input heat reaches the critical stress value of the material itself, the excess heat cannot be released; it can be released only through cracks. With the increase in heat, the width and the number of cracks will gradually increase, making the excess heat quickly spread outward. Xiang, H et al. [29] systematically studied the influence of process parameters on the microstructure of TiN coatings deposited by ESD. The output voltage was too high, and the thermal stress accumulation of the coating was too fast, which may lead to vertical and horizontal microcracks. During the reciprocating contact between the electrode and substrate, the propagation of microcracks may cause the coating to fall off, which is not conducive to the formation of the coating.

In contrast, defects such as cracks or droplets in Figure 4a,d are reduced. With the increase in working capacitance, the thermal shock generated by ESD discharge is enhanced, the thermal stress of the coating is increased, and the brittleness of the TiC coating is increased. Cracks and metal melts appear in the coating during deposition, and the coating defects are increased. Because the difference in thermal expansion coefficient between the brittle coating and the plastic matrix is too significant, cracks will appear at the interface between the coating and matrix under the action of the thermal stress cycle. The production of these defects will reduce the hardness of the coating.

### 3.3. Cross-Sectional Morphology Analysis of TiC Coating on the Surface of AlFeCoCrNiCu High-Entropy Alloy

**Figure 5:**
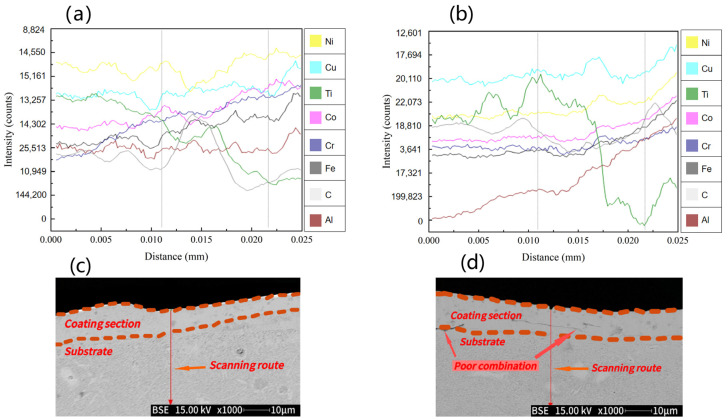
Cross-section of TiC coating: (**a**,**b**) are energy spectrum scans; (**c**,**d**) is a macroscopic morphology.

In order to study the elemental changes in cracks at the boundary between the coating and the substrate, the cross-sectional energy spectrum of the TiC coating of sample 4 was scanned, and the results are shown in Figure 5. Figure 5a shows that energy spectrum line scanning analysis was performed from the coating toward the substrate direction, with a scanning depth of 25 μm. The energy spectrum shows that the distribution of the Fe, Co, Cr, Ni, Cu, and Al elements in the sedimentary layer is uniform and gradually increases. At the boundary, the distribution of the Ti and C elements is uneven and irregularly decreasing. Figure 5c shows that the coating and substrate have a high degree of adhesion and no defects such as cracks. Figure 5b shows the energy spectrum scanning analysis from the coating toward the substrate, with a scanning depth of 25 μm. The energy spectrum shows that the distribution of the Fe, Co, Cr, Ni, and Cu elements in the coating is uniform. However, the content of the Al element is too low, and the Ti and C elements decrease irregularly from the boundary to the substrate. From Figure 5d, it can be seen that there is a clear boundary between the substrate and the coating after 5 s of corrosion with aqua regia. Due to poor adhesion between the coating and substrate, there are some slight cracks at the boundary. This is because the Al element has a larger atomic size than the other elements, and during the solid solution process, the significant size effect promotes the various properties of the coating. Therefore, the interface defects with uniform Al distribution will also be correspondingly reduced. There are also related studies on the Al element in coatings: Cai, Y et al. [30] studied the preparation of FeCoCrNiAlx (x = 0.3, 0.7) coatings using laser cladding technology and found that the Al element can promote lattice transition from FCC to BCC. This improves the microhardness and tribological properties, but reduces corrosion resistance.

### 3.4. Corrosion Behavior of TiC Coating on the Surface of AlFeCoCrNiCu High-Entropy Alloy

#### 3.4.1. Analysis of Potentiodynamic Polarization Test

**Figure 6:**
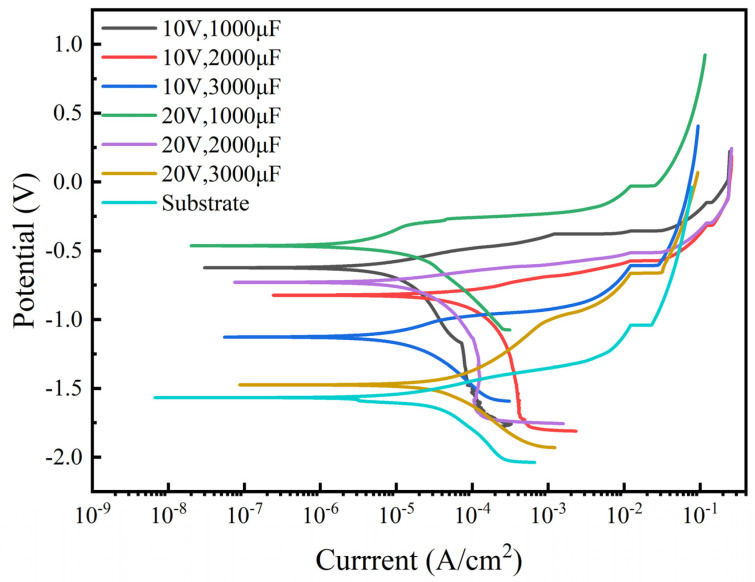
Potential polarization curves of coatings and substrates.

In order to investigate the electrochemical corrosion resistance of TiC coatings and substrates prepared under six different process parameters, the potentiodynamic polarization curves of the coatings and substrates under stable open circuit potential were measured, and the results are shown in Figure 6. From the comparison of dynamic polarization curves, it can be seen that the corrosion resistance of each coating is higher than that of the substrate. The electrochemical parameters of the coating and substrate, including self-corrosion potential (Ecorr), corrosion current density (Icorr), and corrosion rate, were fitted using the Tafel extrapolation method. The results are shown in Table 3. The self-corrosion potential and current corrosion density reflect the material’s corrosion trend and corrosion rate. The higher the self-corrosion potential, the lower the corrosion current density and the better the corrosion resistance [31].

From Table 3, it can be seen that the corrosion current density of sample 4’s coating is the smallest (9.475 × 10^−7^ ± 0.06 × 10^−6^), with the substrate having the highest corrosion current density (7.911 × 10^−6^ ± 0.06 × 10^−6^). The lower the corrosion current density, the slower the corrosion rate. The self-corrosion voltage of all six coatings is higher than that of the substrate; among them, the maximum self-corrosion voltage of sample 4’s coating is −0.445 ± 0.06, much higher than the substrate’s self-corrosion voltage of −1.567 ± 0.06. Therefore, the TiC coatings prepared using six different process parameters have superior corrosion resistance compared with the high-entropy alloy substrates.

#### 3.4.2. Electrochemical Impedance Testing

Electrochemical impedance spectroscopy (EIS) is a method of studying the frequency-dependent relationship of electrochemical impedance by applying a small alternating current excitation signal according to the sine law when an electrochemical cell is in equilibrium (open circuit state) or under a stable, direct-current polarization condition. It is commonly used to investigate the corrosion resistance of metals.

**Figure 7:**
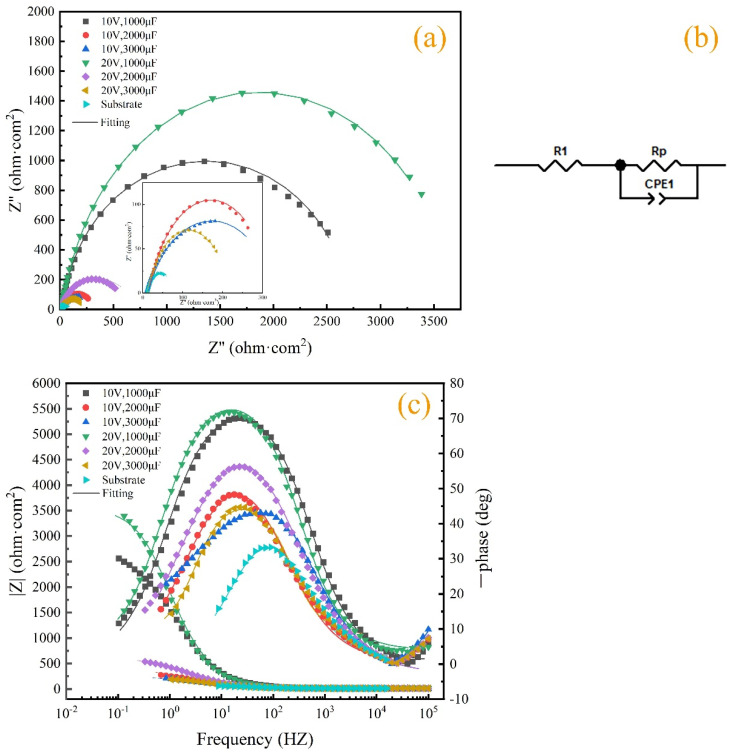
Electrochemical impedance spectroscopy of coatings: (**a**) Nyquist; (**b**) equivalent circuit diagram; (**c**) Bode.

Nyquist plots and Bode plots were obtained by conducting electrochemical impedance tests on TiC coatings and substrates prepared under six different process parameters, as shown in Figure 7. In the Nyquist plot of Figure 7a, six coatings and substrates exhibit a time-constant single capacitance impedance in the corrosive solution. The larger the capacitance arc diameter, the higher the capacitance impedance and the better the corrosion resistance. The equivalent circuit diagram in Figure 7b shows Rs is the solution resistance and R1 is the charge transfer resistance. Due to certain roughness on the coating surface, it is necessary to introduce CPE long positional angle components to replace double-layer capacitors [32]. Figure 7c shows the Bode plot of the samples after corrosion in a 3.5% wt NaCl solution. Among them, it can be concluded from Figure 7c that the coating impedance of sample 4 is the highest, at 2.502 × 10^3^ Ω·com^2^. From Figure 7c, it also can be concluded that sample 4 has the highest absolute value of phase angle (approximately 72°). The results show that the samples with deposited coatings had better corrosion resistance than the substrate, and sample 4 had the best corrosion resistance with the coating.

The equivalent circuit diagram used for fitting analysis is shown in Figure 7b, where Rs represents the resistance in the electrolyte; R_P_, in series with Rs, represents the equivalent polarization resistance in the electrode bilayer; CPE is connected in parallel with the equivalent polarization resistance (Rs) to represent the capacitance in the circuit. The data obtained from the fitting analysis are shown in Table 4. The larger the R_P_, the better the wear resistance of the coating. The closer CPE1-P is to 1.0, the denser and more uniform the passivation film on the coating surface [33]. The maximum R_P_ value of the coating of sample 4 is 3688 Ω·com^2^, and the minimum R_P_ value of the substrate is 67.13 Ω·com^2^. The CPE1-P of the coating of sample 4 is closest to 1.0, about 0.85955. The results show that the corrosion resistance of the six coatings was better than that of the substrate, and the corrosion resistance of the sample 4 coating was the best.

In summary, the corrosion resistance of the TiC coating prepared using spark deposition technology is superior to that of the substrate, and the TiC coating of sample 4 has the best corrosion resistance. Cao, G et al. [34] prepared Cr coatings with different parameters (100 V–60 μF, 100 V–90 μF, 100 V–120 μF, 150 V–60 μF, 150 V–90 μF, and 150 V–120 μF) on the surface of M50 steel using electrical discharge deposition technology. The influence of the deposition process parameters on the corrosion resistance of the coatings was studied. The research found that the surface of the coating presents a splash-like pattern accompanied by cracks. The corrosion resistance of the coating was significantly better than that of the substrate, and the coating deposited under the conditions of 150 V–60 μF had the most negligible defect density and the best corrosion resistance. This experimental result is similar to our current experimental result. The influence of voltage on deposition rate is more significant than that of capacitance because as the voltage increases, the pulse energy increases, the melting rate and deposition rate of the electrode material increase, and the limit of mass gain increases. As the capacitance increases, the pulse energy, thermal residual stress, and impact effect increase. This leads to a decrease in the deposition rate of the electrode materials [35].

The TiC coating prepared on the surface of the AlFeCoCrNiCu high-entropy alloy block using electric discharge deposition inevitably exhibits defects such as cracks and metal droplets. During the corrosion process, corrosion initially occurs on defects such as pores and microcracks on the coating surface, and it gradually spreads to the coating surface. However, the surface area of cracks is generally smaller than that of coatings, which means that the corrosion current is concentrated in small areas. Hence, the corrosion current in areas with cracks is higher. As shown in Figure 4f, sample 6, prepared with the maximum operating voltage and current (20 V–3000 μF), has the most significant surface cracks and the highest corrosion current among the six coatings, 6.901 × 10^−6^ ± 0.06 × 10^−6^. Therefore, the TiC coating on the surface of sample 6 has the worst corrosion resistance. Combining the electrochemical corrosion results with Figure 3 and Figure 4, it is not difficult to observe that defects such as cracks on the coating surface increase as the operating voltage and current increase. The occurrence of defects reduces the hardness of the coating, and the appearance of cracks reduces its corrosion resistance.

## 4. Conclusions

This article investigates the effects of operating voltage and current on the corrosion resistance and hardness of TiC coatings prepared on the surface of AlFeCoCrNiCu high-entropy alloy using three different process parameters. The research results are as follows:

1. The microhardness of the TiC coating is higher than that of the substrate. The microhardness of sample 4 is 844.98 HV, which is 81% higher than that of the substrate. This indicates that a TiC coating can improve the microhardness of the substrate surface and enhance its mechanical properties. Moreover, with the increase in capacitance and output voltage, the oxidation of the TiC coating intensifies, and the oxidation product TiO_2_ increases, which will cause a decrease in the hardness of the TiC coating.
2. By comparing the surface morphology of six different coatings, it was found that samples with smaller working capacitance had fewer surface defects on the coating. This is because the working capacitance is too large, which increases the thermal stress of the coating, leading to an increase in the brittleness of the TiC coating. During the deposition process, cracks and molten metal appear in the coating, increasing coating defects. Under the action of thermal stress cycling, cracks may also appear at the interface between the coating and the substrate.
3. Energy spectrum line scanning analysis was conducted at the boundary between the coating and the substrate, and it was found that the change in the content of the Al element in the coating plays a crucial role in the bonding between the coating and the substrate. This is because the Al element has a larger atomic size compared with the other elements. During the solid solution process, the significant size effect promotes the various properties of the coating.
4. Action potential tests were conducted on TiC coatings and high-entropy alloy substrates prepared under six different process parameters. The corrosion current density of the sample 4 coating was the smallest (9.475 × 10^−7^ ± 0.06 × 10^−6^). After conducting electrochemical impedance testing, it was found that the maximum RP value of the coating on sample 4 was 3688 Ω·com^2^, and the overall trend of the dynamic potential test results was the same.

## Figures and Tables

**Table 1:** Nominal chemical compositions of AlFeCoCrNiCu high-entropy alloys (wt.%).

Element	Al	Fe	Co	Cr	Ni	Cu
Content	9.59	15.12	17.54	18.25	19.70	19.81

**Table 2:** Process parameters of electric discharge deposition.

Sample	Voltage/V	Capacitance/μF
1	10	1000
2	10	2000
3	10	3000
4	20	1000
5	20	2000
6	20	3000

**Table 3:** The potentiodynamic polarization parameters of the sample in a 3.5% NaCl solution.

	E_corr_ (V_SHE_)	I_corr_ (A·cm^−2^)
Substrate	−1.567 ± 0.06	7.911 × 10^−6^ ± 0.06 × 10^−6^
10 V, 1000 μF	−0.623 ± 0.06	1.087 × 10^−6^ ± 0.06 × 10^−6^
10 V, 2000 μF	−0.837 ± 0.06	4.417 × 10^−6^ ± 0.06 × 10^−6^
10 V, 3000 μF	−1.126 ± 0.06	6.338 × 10^−6^ ± 0.06 × 10^−6^
20 V, 1000 μF	−0.445 ± 0.06	9.475 × 10^−7^ ± 0.06 × 10^−7^
20 V, 2000 μF	−0.730 ± 0.06	1.239 × 10^−6^ ± 0.06 × 10^−6^
20 V, 3000 μF	−1.465 ± 0.06	6.901 × 10^−6^ ± 0.06 × 10^−6^

**Table 4:** Electrochemical impedance parameters of samples in 3.5% NaCl solution.

	R_s_/(Ω·com^2^)	R_P_/(Ω·com^2^)	CPE1-T	CPE1-P
Substrate	10.77	67.13	3.8405 × 10^−4^	0.73933
10 V, 1000 μF	10.76	2626	9.2256 × 10^−5^	0.84314
10 V, 2000 μF	13.05	318.7	4.4542 × 10^−4^	0.74324
10 V, 3000 μF	9.22	253	5.7101 × 10^−4^	0.67879
20 V, 1000 μF	13.02	3688	8.9813 × 10^−5^	0.85955
20 V, 2000 μF	10.57	596.3	2.8713 × 10^−4^	0.77062
20 V, 3000 μF	12.39	217.2	4.44667 × 10^−4^	0.73698

## Data Availability

The data presented in this study are available on request from the corresponding author.
